# *Epilepsia Open*—December 2024 announcements

**DOI:** 10.1002/epi4.13109

**Published:** 2024-12-11

## ILAE Congresses

### 7th East Mediterranean Epilepsy Congress

12–15 December 2024

Baghdad, Iraq

### ASEPA Neuropsychology Course in Epilepsy 2024

14–15 December 2024

Taipei City, Taiwan

## 2025

### Epilepsy Workshop

16–17 January 2025

Egypt

### 14th ILAE School on Pre‐Surgical Evaluation for Epilepsy and Epilepsy Surgery

20–24 January 2025

Brno, Czech Republic

### 15th Asian & Oceanian Epilepsy Congress

20–23 February 2025

New Delhi, India

### 18th Latin American Summer School on Epilepsy

7–15 March 2025

São Paulo, Brazil

### 7th ILAE School on EEG in the First Year of Life

26–27 April 2025

Sanya City, Hainan, China

### ILAE School on Neuroimaging 2025

15–17 May 2025

Potsdam, Germany

### 5th African Epilepsy Congress

16–18 May 2025

Lusaka, Zambia

### XVIII Workshop on Neurobiology of Epilepsy (WONOEP 2025)

25–29 August 2025

Portugal

### 36th International Epilepsy Congress

30 August–3 September 2025

Lisbon, Portugal

## 2026

### 16th European Epilepsy Congress

5–9 September 2026

Athens, Greece

## ILAE Webinars

### Barriers to Care Pathways

3 December 2024

### Self Limiting Epilepsy

6 December 2024

### Acute Management of Functional/Dissociative Seizures

16 January 2025

## Other Congresses

### Encephalitis 2024

2–3 December 2024

London, UK & Online

### Second Symposium for Libyan Scientific Society for Epilepsy

6–7 December 2024

Tripoli, Libya

### AES 2024 Annual Meeting

6–10 December 2024

Los Angeles, California, USA

### IBRO/ILAE Epilepsy School

9–12 December 2024

Kinshasa, Democratic Republic of Congo

### In Search of Lost Time 5

16–18 December 2024

Rome, Italy

## 2025

### 1er Congreso Latinoamericano de Epilepsias Genéticas 2025

8–10 January 2025

Santiago, Chile

### 3rd International Online Course on Pathogenesis of Epilepsy

27 February–29 May 2025

Online

### World Neuroscience and Psychiatry Conference 2025 (WNPC25)

8–9 March 2025

Bangkok, Thailand

### 19th World Congress on Controversies in Neurology

20–22 March 2025

Prague, Czech Republic

### 13th Trinational Conference of the German and Austrian Society for Epileptology and the Swiss Epilepsy League

26–29 March 2025

Salzburg, Austria

### AD/PD™ 2025 International Conference on Alzheimer's and Parkinson's Diseases and related neurological disorders

1–5 April 2025

Vienna, Austria & Online

### International Congress on Structural Epilepsy & Symptomatic Seizures 2025

2–4 April 2025

Gothenburg, Sweden

### 12th International Residential Course on Drug Resistant Epilepsies

11–27 May 2025

Tagliacozzo, Italy

### International Conference for Technology and Analysis of Seizures (ICTALS 2025)

2–6 June 2025

Montreal, Canada

### 11th Congress of the European Academy of Neurology

21–25 June 2025

Sevilla, Spain

### 21st San Servolo Advanced Epilepsy Course

21 July–1 August 2025

San Servolo (Venice), Italy

### 19th European Congress for Clinical Neurophysiology

9–12 September 2025

London, UK

## 2026

### 18th Eilat Conference on New Antiepileptic Drugs and Devices

3–6 May 2026

Madrid, Spain

